# Ubiquitin-modifying enzymes in Huntington’s disease

**DOI:** 10.3389/fmolb.2023.1107323

**Published:** 2023-02-08

**Authors:** Karen A. Sap, Karlijne W. Geijtenbeek, Sabine Schipper-Krom, Arzu Tugce Guler, Eric A. Reits

**Affiliations:** Department of Medical Biology, Amsterdam UMC, University of Amsterdam, Amsterdam, Netherlands

**Keywords:** Huntington’s disease, huntingtin, ubiquitin, neurodegenerative disease, ligase, deubiquitinating enzyme, proteostasis, proteasome

## Abstract

Huntington’s disease (HD) is a neurodegenerative disorder caused by a CAG repeat expansion in the N-terminus of the HTT gene. The CAG repeat expansion translates into a polyglutamine expansion in the mutant HTT (mHTT) protein, resulting in intracellular aggregation and neurotoxicity. Lowering the mHTT protein by reducing synthesis or improving degradation would delay or prevent the onset of HD, and the ubiquitin-proteasome system (UPS) could be an important pathway to clear the mHTT proteins prior to aggregation. The UPS is not impaired in HD, and proteasomes can degrade mHTT entirely when HTT is targeted for degradation. However, the mHTT protein is differently ubiquitinated when compared to wild-type HTT (wtHTT), suggesting that the polyQ expansion affects interaction with (de) ubiquitinating enzymes and subsequent targeting for degradation. The soluble mHTT protein is associated with several ubiquitin-modifying enzymes, and various ubiquitin-modifying enzymes have been identified that are linked to Huntington’s disease, either by improving mHTT turnover or affecting overall homeostasis. Here we describe their potential mechanism of action toward improved mHTT targeting towards the proteostasis machinery.

## 1 Introduction

### 1.1 Huntington’s disease

Huntington’s disease (HD) is an inherited neurodegenerative disease caused by a CAG repeat expansion in exon1 of the Huntingtin (*HTT*) gene (MacDonald et al., 1993). The CAG repeat expansion results in a polyglutamine (polyQ) expansion in the HTT protein. N-terminal fragments of the mutant HTT (mHTT) proteins containing the polyQ repeat are aggregation-prone and form intracellular inclusion bodies (IBs) (DiFiglia et al., 1997), as observed in human HD post-mortem brain and in animal or cellular systems. A repeat length of 40 repeats and longer results in HD, whereby the CAG repeat length is inversely correlated with age of onset (Fusilli et al., 2018). The symptoms of HD comprise motor- and cognitive decline and psychological deficits, which aggravate over time, and are lethal approximately 15 years after the onset of the disease. There is no cure for HD. Lowering mHTT by improving its degradation prior to aggregation and toxicity would be a therapeutic strategy to prevent, delay or slow down the onset and progression of disease. The two main intracellular pathways involved in protein degradation are the ubiquitin-proteasome system (UPS) and autophagy. Both pathways play a role in mHTT clearance (Rubinsztein, 2006; Thompson et al., 2009). Although the UPS is active in both the nucleus and the cytoplasm, it is merely capable of degrading unfolded monomeric HTT proteins (Waelter et al., 2001; Li et al., 2010). The autophagic pathway is a cytoplasmic degradation machinery and targets soluble and aggregated HTT proteins for lysosomal destruction (Ravikumar, 2002). The proteasome is not impaired by mHTT fragments, as initially thought, but can degrade mHTT entirely when efficiently targeted towards proteasomes *via* ubiquitination, and the proteasome can degrade the polyQ sequence itself (Juenemann et al., 2013). In addition, both the proteasome (Schipper-Krom et al., 2014) and ubiquitin (Juenemann et al., 2018) are not sequestered into mHTT aggregates but are dynamically and reversibly recruited. Together, this indicates that the UPS is not impaired in cells expressing mHTT and that efficient targeting of mHTT to proteasomes results in complete degradation. So why is mHTT not efficiently degraded? Recent publications show that the polyQ expansion affects the ubiquitination of mHTT, as shown in different HD mouse and rat models (Sap et al., 2019; Hakim-Eshed et al., 2020), with differences in ubiquitination sites between wtHTT and mHTT. Both HTT degradation and aggregation have been associated with several ubiquitin-modifying enzymes, and we will describe the various ubiquitinating and de-ubiquitinating enzymes that may play a role in mHTT degradation.

### 1.2 Ubiquitination as a post-translational modification

Ubiquitin is a highly conserved small (8.5 kDa) modular signaling protein that functions as a post-translational modification and can be used to target proteins for degradation. Ubiquitin is attached mainly to internal lysine residues in target proteins (Hershko and Ciechanover, 1998). Additionally, ubiquitin can be attached to target proteins’ N-terminal methionine (M1) (Breitschopf et al., 1998). Ubiquitin can also be a substrate for ubiquitination, where the amino acids M1, K6, K11, K27, K29, K33, K48, and K63 of the ubiquitin protein serve as target residues for polyubiquitin chain formation (Swatek and Komander, 2016). Polyubiquitination with the same linkage type results in polyubiquitin chains, while polyubiquitin branches are formed with mixed linkage types. Polyubiquitin chains and branches formed through K48 and K11 are the “canonical” degradation signals (Jacobson et al., 2009; Min et al., 2015), whereas K63 polyubiquitination plays a role in signaling, protein trafficking, aggregate formation, and autophagy (Tan et al., 2008; Chen and Sun, 2009). Recent insights suggest a “ubiquitination threshold” model for proteasomal degradation where multiple short K48-linked chains or branched structures with K11 and K48 linkages are efficient degradation signals. In this model, the amount of polyubiquitin plays a more important role than the type of polyubiquitin linkage (Swatek and Komander, 2016).

### 1.3 Ubiquitin-modifying enzymes

**FIGURE 1:**
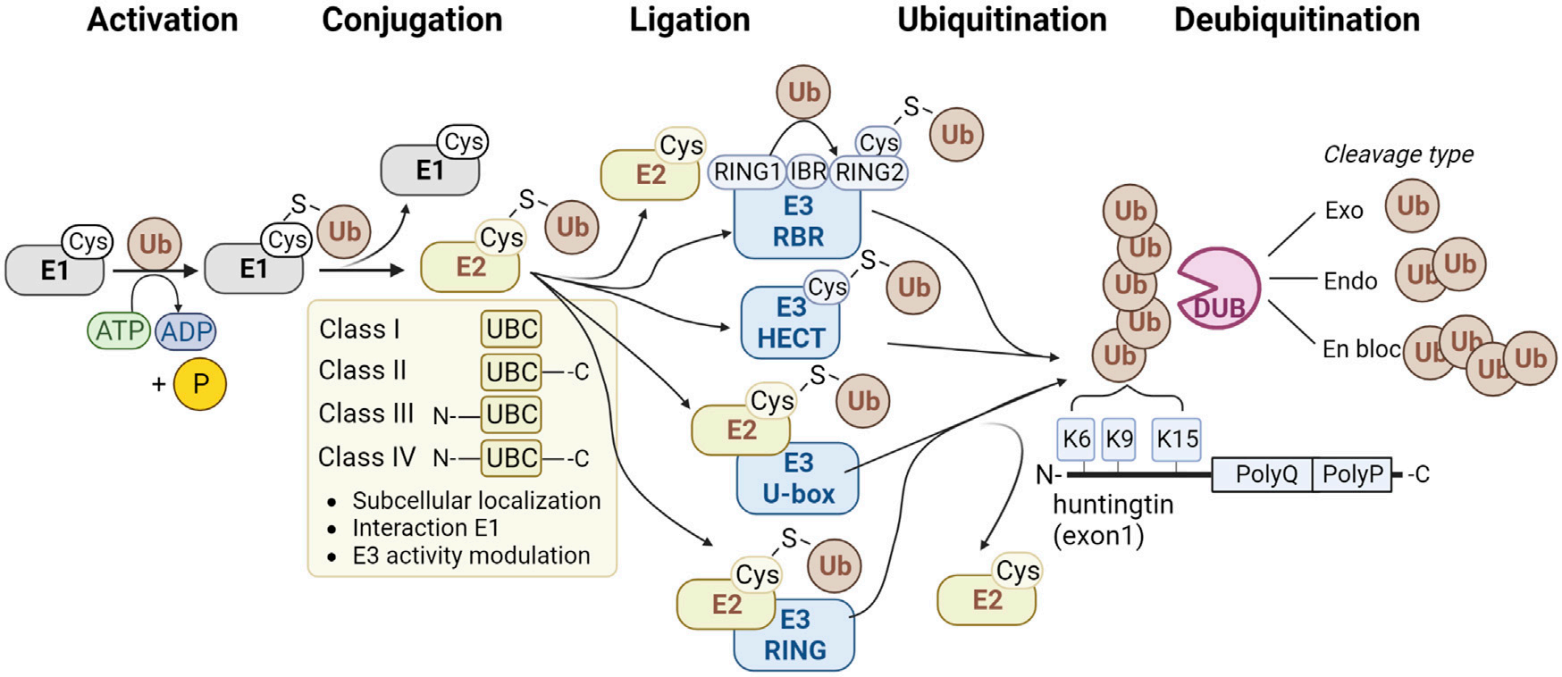
Method of action of enzyme classes involved in the ubiquitination pathway. The classes of E1 ubiquitin-activating enzymes are shown (grey), four classes of E2 ubiquitin-conjugating enzymes (yellow), and four families of E3 ubiquitin ligases (blue) and deubiquitinating enzymes (pink). Ubiquitination sites K6, K9 and K15 at the N-terminus of HTT are shown at the right.

Ubiquitin is attached to target proteins through the sequential action of E1 ubiquitin-activating enzymes, E2 ubiquitin-conjugating enzymes, and finally, E3 ubiquitin ligases (Figure 1). Firstly, the E1 enzyme activates the ubiquitin molecule by forming a thiol ester bond between its active site cysteine and the C-terminus of the ubiquitin molecule in an ATP-dependent manner. The E1 enzyme then transfers the activated ubiquitin molecule to the active site cysteine of an E2 enzyme. Subsequently, the E2 enzyme forms a complex with an E3 ubiquitin ligase. The E3 enzyme binds to the target protein and transfers ubiquitin directly or indirectly from the E2 enzyme to the target protein. There are 2 E1, ∼40 E2, and ∼600 E3 enzymes in the ubiquitin pathway (Clague et al., 2015). Deubiquitinating enzymes (DUBs) can remove ubiquitin. There are approximately 100 DUBs (Clague et al., 2019), including DUBs that can remove complete polyubiquitin chains *en bloc*, DUBs that cleave within chains, and DUBs that remove single ubiquitin molecules from the tip of the polyubiquitin chain (Figure 1).

#### 1.3.1 E1 ubiquitin-activating enzymes

The E1 family consists of eight members in human, of which two can activate ubiquitin, and the others activate different Ubiquitin-like (UBL) proteins. UBA1 and UBA6 can activate ubiquitin, and contain an N-terminal adenylation domain to bind ATP and ubiquitin, a catalytic cysteine domain, and a C-terminal ubiquitin-fold domain for E2 recruitment. E1 enzymes function on the apex of the ubiquitination pathway to activate ubiquitin with the use of ATP and transfer it to the E2 enzyme (Figure 1). UBA1 seems to play a more general role in activating ubiquitin compared to UBA6. UBA1, for instance, is ∼ ten times more abundant than UBA6 (Yang et al., 2013; Kulak et al., 2014), and this pool is fully charged with ubiquitin, while UBA6 is charged for ∼50% and shows specificity for E2 enzyme UBE2Z/USE1 (Jin et al., 2007) which is involved in targeting proteins with destabilized N-terminal amino acids for degradation (Lee et al., 2011).

#### 1.3.2 E2 ubiquitin-conjugating enzymes

The human genome comprises approximately 40 E2 enzymes (Y. Ye and Rape, 2009). All E2s share a common catalytic core domain, *i.e.*, the Ubiquitin Conjugation (UBC) domain (Wijk and Timmers, 2010), which forms a thioester bond with ubiquitin. The E2 superfamily is divided into four classes (Figure 1). Class I E2 enzymes only contain the UBC domain, while class II E2 enzymes contain the UBC domain plus a C-terminal extension. The class III E2 enzymes contain the UBC domain and an N-terminal extension. Class IV E2 enzymes contain a UBC domain plus an N- and C-terminal extension (Burroughs et al., 2008; Michelle et al., 2009). The different classes show functional diversity and can affect the subcellular localization or modulate the interaction with E1 and E3 enzymes. There is some cross-reactivity among E2 and E3 enzymes, as the same E3 can interact with various E2 enzymes and *vice versa*, which could be RING and HECT enzymes. Structural characteristics and function of E2 enzymes are reviewed elsewhere in more detail (Wijk and Timmers, 2010).

#### 1.3.3 E3 ubiquitin ligases

E3 ubiquitin ligases are enzymes that recognize substrates that need to be ubiquitinated. Based on differences in structure, E3 ubiquitin ligases can be divided into four types: HECT type, RING-finger type, U-box type, and RBR type (Figure 1).

##### 1.3.3.1 HECT E3 ligases

The human HECT E3 ligase family consists of 28 members, which all vary in their N-terminal domain, used for substrate recognition (Sluimer and Distel, 2018), while they all share a conserved homologous to E6-AP C-terminus domain, or HECT domain, used to bind ubiquitin. Based on the types of N-terminal domains, the HECT E3 ligases can be divided into three major subfamilies: the NEDD4 subfamily with nine members, the HERC subfamily with six members, and the group of ‘other HECTs’ with thirteen members (Rotin and Kumar, 2009). The regulation of the HECT E3 enzymes has been reviewed elsewhere (Sluimer and Distel, 2018). HECT E3 ligases receive a ubiquitin molecule from an E2 enzyme. Next, the ubiquitin-loaded HECT E3 enzyme interacts with the substrate proteins *via* their N-termini and transfers ubiquitin to the substrates. In contrast, RING-finger E3 ligases do not receive the ubiquitin molecule from the E2 enzyme. RING-finger E3 ligases are described below.

##### 1.3.3.2 RING-finger E3 ubiquitin ligases

The Really Interesting New Gene (RING)-finger E3 ubiquitin ligases comprise, with over 600 members, the largest family of E3 ubiquitin ligases. RING-finger E3s share a RING domain, which is used to interact with E2 enzymes, but this domain does not interact with ubiquitin as it lacks a *bona fide* catalytic center. Instead, the ubiquitin molecule is transferred directly from the E2 onto the substrate. RING-finger E3 ubiquitin ligases thus function as scaffolds between the E2 and the substrate. Many variations exist between RING-finger E3s as they could function as monomeric proteins but could also be part of large multi-subunit complexes, such as cullin-RING ligases and the anaphase-promoting complex/cyclosome (APC/C) complex. Also, the substrates of RING-finger E3s are highly varied, as they might recognize multiple different substrates, and various E3 ubiquitin ligases could also recognize a specific substrate. The ligase activity and substrate recognition can be modulated *via* post-translational modifications. RING-type E3 ligases have been reviewed in more detail elsewhere (Metzger et al., 2014).

##### 1.3.3.3 U-box E3 ligases

The number of U-box ubiquitin ligases varies widely per species, with 21 U-box proteins in humans, 101 in Chinese cabbage *B. rapa* (Wang et al., 2015) and 125 in soybean *Glycine max* (Wang et al., 2016). The C-terminal U-box domain is conserved from yeast to humans and has a three-dimensional structure similar to the RING-finger domain but lacks the metal chelating residues (Aravind and Koonin, 2000). Similar to RING-finger ubiquitin ligases, the U-box domain is used to interact with E2s, and ubiquitin is directly transferred from the E2 enzyme to the substrate. U-box E3 ligases are often involved in polyubiquitination and some can elongate polyubiquitin chains and are also called E4 ubiquitin ligases. Also, some U-box E3s interact with chaperones to play a role in the protein quality control system. U-box type E3 ubiquitin ligases are reviewed in more detail elsewhere (Hatakeyama and Nakayama, 2003).

##### 1.3.3.4 RBR E3 ligases

Finally, the fourth type of E3 ubiquitin ligases comprises the RING-between-RING (RBR) E3s, and this family has 14 members in humans. RBR ligases contain a RING1 domain, followed by a central in-between-RINGs (IBR) domain and a RING2 domain. The RING1 domain recruits the ubiquitin-loaded E2 and transfers ubiquitin to the catalytic cysteine in the RING2 domain, which transfers it to the substrate (Spratt et al., 2014). Since RBR ligases contain two RING domains but a HECT-type E3 mechanism of work, these enzymes are also called RING-HECT hybrids. Examples of RBR E3 ligases are Parkin (PRKN), involved in Parkinson’s disease, and HOIP, a member of the linear ubiquitin chain assembly complex (LUBAC), responsible for assembling M1-linked linear Ub chains. RBR ligases have been reviewed in more detail elsewhere (Walden and Rittinger, 2018).

#### 1.3.4 Deubiquitinating enzymes

The ubiquitination of protein substrates like HTT becomes reversible through the action of deubiquitinating enzymes (DUBs), which can cleave off ubiquitin in two ways. They can either recognize and target specific protein interaction domains or show specificity for particular polyubiquitin linkage types. The latter type of DUB may hydrolyze polyubiquitin chains but leave the proximal ubiquitin on the substrate. Linkage specificity is determined by the catalytic domain, ubiquitin-binding domains, or by the specificity of interacting proteins. Apart from linkage selectivity, DUBS could also show selectivity in removing off ubiquitin moieties from the end of polyubiquitin chains or cleaving within chains. There are seven different families of DUBs. Six of them are classified as cysteine proteases: USPs, UCHs, OTUs, MJDs (also known as Josephins), MINDYs, and ZUP1. The seventh family is the JAMM family, also known as MPN, and is classified as metalloproteinase. DUB specificity and regulation have been reviewed elsewhere in more detail (Mevissen and Komander, 2017).

### 1.4 HTT ubiquitination sites

Aberrant splicing and protein cleavage can generate different mHTT protein fragments, including the N-terminal exon1 fragment containing the polyQ expansion. These fragments were shown to be highly pathogenic in HD mouse models and are also observed in fibrillar aggregates in brains of HD patients (DiFiglia et al., 1997; Schilling et al., 2007; Landles et al., 2010). The mHTT fragments can be present as monomers, soluble oligomers, or insoluble aggregates, including the insoluble IBs. Especially soluble oligomeric mHTT species appear to be most toxic to the cell. IBs seem to have a protective role by sequestering toxic oligomeric mHTT species (Arrasate et al., 2004). However, IB formation also results in the sequestration of other proteins from their normal cellular environment, thereby interfering with important processes, including transcriptional regulation (Schaffar et al., 2004) and proteostasis (Park et al., 2013). The N-terminus of the HTT exon1 contains 17 amino acids, including three lysine residues, followed by the polyQ region and a polyproline region (Finkbeiner, 2011). Interestingly, various post-translational modifications (PTMs) of the N17 domain of HTT have been described, including acetylation, SUMOylation, and ubiquitination at lysines 6, 9, and 15 and phosphorylation at threonine 3, serine 13 and serine 16 that affect aggregation, toxicity and degradation (Steffan et al., 2004; Thompson et al., 2009; Maiuri et al., 2013; DeGuire et al., 2018). Within full-length HTT, most PTMs can be found in clusters within predicted unstructured domains but not in the structured HEAT repeats (Arbez et al., 2017). When compared to the many identified phosphorylation and acetylation sites, only nine ubiquitination sites (K6, K9, K15, K132, K337, K631, K804, K837, and K2097) have been identified for HTT (Steffan et al., 2004; Juenemann et al., 2015; Yau et al., 2017; Koyuncu et al., 2018; Sap et al., 2019; Hakim-Eshed et al., 2020). Ubiquitination at K6 and K9 was specific for mHTT in mice and rat brains (Sap et al., 2019; Hakim-Eshed et al., 2020), indicating that polyQ expansion in the HTT protein affects ubiquitination. Differential ubiquitination of wtHTT and mHTT could be the result of different interactions and affinities for (de) ubiquitinating enzymes. In addition, both the proteome and ubiquitinome are changed, including numerous components of the involved proteostasis pathways (Sap et al., 2019). Expression of mHTT exon1 lacking these ubiquitination sites in cells was found to slow down aggregation and reduce IB size. However, it increased both toxicity and the number of much smaller aggregates, suggesting that ubiquitination of the N17 domain of HTT by yet unidentified enzymes affects both aggregation and toxicity (Hakim-Eshed et al., 2020). Studies focusing on soluble and insoluble HTT ubiquitination have been previously reviewed in more detail (Sap and Reits, 2020).

## 2 Ubiquitin-modifying enzymes linked to HD

Several ubiquitin ligases and DUBs have been linked to HD. These enzymes might be potential therapeutic targets to facilitate the degradation of mHTT by the proteostasis machinery prior to aggregation. Here we give some background information about the enzymes and describe how these enzymes were linked to HD. For instance, the enzymes might affect proteasome-dependent degradation of mHTT, or affect solubilization, polymerization, or aggregation of mHTT. Eventually, the ubiquitin-modifying enzymes could affect cellular pathways and cellular stress that affect HD pathogenicity directly or indirectly. We also describe whether the ubiquitin-modifying enzymes might be a potential target for HD therapy. We start with discussing the involved E1 enzyme, followed by E2 enzymes, and finally E3 enzymes, which are categorized by type, starting with HECT E3s, followed by RING-finger E3s, RBR E3, and finally U-box E3s. The DUBs are described per class and USP type of DUBs in alphabetical order. The effects of these ubiquitin-modifying enzymes on mHTT levels, interactions, aggregation and pathology are summarized in Table 1 and in more detail in Supplementary Table S1. Finally, we discuss difficulties in comparing HD studies, based on different mHTT Q-lengths, fragment lengths, and tags used, but also different methods available to study soluble and insoluble mHTT protein levels and different models of disease.

**TABLE 1:** Effects of ubiquitin-modifying enzymes on cellular mHTT and HD pathology.

		Subcellular localization	Model*	Enzyme levels in HD	Interaction or colocalization	Enzyme modulation	Pathology	IBs	HTT protein levels mono/polymer	Reference
E1	UBA1	Cytosol, nucleoplasm	Mouse	↓		Inh. PYR41			↑ (Poly)	Wade et al. (2014)
			Mouse—flHTT			Inh. PYR41			↑ (Poly)	
E2	HIP2	Cytosol, plasma membrane	*in Vitro*	↑	yes					Kalchman et al. (1996)
			SH-SY5Y		yes	OE		No effect		de Pril et al. (2007)
						OE mutant	↓	↓		
						RNAi	↓	↓		
			HD patient iPSCs			RNAi		No effect		Koyuncu et al. (2018)
	UBE2W	Nucleoli	HEK293			OE		↑	No change (Mono/Poly)	Wang et al., 2018
						OE mutant^1^		↓	No change (Mono); ↓(Poly)	
						OE mutant^2^		↓	↑ (Mono); ↓ (Poly)	
			Neurons			KO	↓	↓	↓ (Total levels)	
			Mouse—flHTT			KO	No effect	No effect	↑ (Mono); ↓ (Poly)	
E3	UBE3A	Cytosol, nucleoplasm	Mouse—flHTT	↓	yes	OE		↓		Bhat et al. (2014)
			HEK293		yes	OE			↓ (Mono)	
			HEK293—flHTT			RNAi			↑ (Poly)	
			Mouse		yes					Maheshwari et al. (2012)
			N2a		yes					Mishra et al. (2009)
			N2a	↑	Yes	OE	↓	↓	↓ (Poly)	Mishra et al. (2008)
						OE mutant		No effect		
						RNAi		↑	↑ (Poly)	
			Mouse		Yes					
			Mouse			KO	↑	↑		Maheshwari et al. (2014)
			HD patient iPSCs			RNAi		No effect		Koyuncu et al. (2018)
	UBR5	Cytosol, nucleoplasm	HD patient iPSCs			RNAi		↑	↑ (Mono)	Koyuncu et al. (2018)
			HEK293			OE		↓	↓ (Mono)	
						OE mutant		No effect	No effect	
	WWP1	Cytosol, Golgi, plasma membrane	Mouse	↑	Yes					Lin et al. (2016)
			N2a	↑	Yes	OE	↑	↑	↑ (Mono)	
						RNAi	↓	↓	↓ (Mono)	
	HACE1	ER, nuclear bodies	STHdh—flHTT	↓		OE	↓			Rotblat et al. (2014)
			Human brain	↓						
			Mouse—flHTT			KO	↑			Ehrnhoefer et al. (2018)
	TRAF6	Mitochondria, nucleoli	HEK293		Yes	OE		↑	No effect	Zucchelli et al. (2011)
						OE mutant		No effect		
			Mouse		Yes					
			Human brain	↑	Yes					
	PJA1	Nucleoplasm, nucleoli	N2a	↓		RNAi		↑	↑ (Mono)	Ghosh et al. (2021)
			HEK293		Yes	OE		↓	↓ (Total levels)	
			Neurons		Yes	OE		↓	↓ (Poly)	Watabe et al. (2022)
						OE mutant			No effect	
			Human brain		No					
	UHRF2	Nucleoplasm	HeLa (NLS)			OE			No effect (Mono); ↓ (Poly)	Iwata et al. (2009)
						OE mutant			No effect	
						OE				
						OE mutant			No effect	
					Yes	OE	↓			
						RNAi	↑	↑	↑ (Poly)	
			Mouse		Yes					
			Neurons (NLS)			OE	↓			
						RNAi	↑			
	San1p–closest yeast homolog of UHRF-2	Nucleus, cytoplasm^¶^	*S. cerevisiae* (NLS)			OE			↓ (Total levels)	
						OE mutant			No effect	
			HeLa (NLS)			OE		↓	↓ (Poly)	
						OE mutant		No effect	No effect	
						OE				
						OE mutant			No effect	
						OE	↓			
	HRD1	Nucleoplasm, ER, plasma membrane	HEK293	↑	Yes	OE		↓		Yang et al. (2007)
						OE mutant				
						RNAi			↑ (Mono)	
			SH-SY5Y			OE			↓ (Mono)	
						OE mutant			No effect	
					Yes	OE	↓	↓	↓ (Mono/Poly)	
						OE mutant	No effect			
	PRKN	Nuclear speckles, cytosol	Patient skin fibroblasts	↑						Aladdin et al. (2019)
			Mouse		Yes					Tsai et al. (2003)
			Human brain		Yes					
			Mouse			KD	↑	↓		Rubio et al. (2009)
	HOIP	Cytosol	SH-SY5Y		Yes	OE		↓		Well et al. (2019)
						OE mutant		↑		
						RNAi	↑	↑		
			HEK293		Yes					
			Mouse	↓	Yes					
			Human brain	↓	Yes					
	CHIP	Cytosol, nucleoplasm	Cos-7			OE		↓	↑ (Mono)	Miller et al. (2005)
						OE mutant^2^			No effect	
			Mouse			KD	↑	↑		
			N2a		Yes	OE	↓	↓		Jana et al. (2005)
						OE mutant	No effect	No effect		
			Neurons -exon1	No change	No					Zhao et al. (2017)
			Astrocytes	↑	Yes	RNAi			↑ (Mono); ↑ (Poly)	
			Mouse (neurons)		No					
			Mouse (astrocytes)		Yes	RNAi				
			Drosophila			OE	↓			Al-Ramahi et al. (2006)
	SKP1	Cytosol, nucleoplasm	Mouse	↓						Bhutani et al. (2012)
			Drosophila			RNAi	↑			
	CUL1	Nucleoplasm, nucleoli	Mouse	↓	No					Bhutani et al. (2012)
			N2a	↓	No	Neg. OE^§^		↑		
			Drosophila	↓		RNAi	↑			
DUB	ATXN3	Nucleoplasm, nucleoli, plasma membrane	Mouse—flHTT		No	KO	↑	No effect	No effect	Zeng et al. (2013)
			Mouse		Yes					Gao et al. (2019)
			SH-Sy5Y—endogenous		Yes					
			PC12		Yes					
	OTULIN	Mitochondria, plasma membrane	SH-SY5Y			RNAi	↓			Well et al. (2019)
	YOD1	Nucleus, plasma membrane, cytosol	SH-SY5Y	↑		OE	↓		↓ (Mono)	Tanji et al. (2018)
						OE mutant	No effect			
			Human brain		No					
	USP7	Nucleoplasm, nuclear bodies	Mouse		Yes					Pluciennik et al. (2021)
			HD patient iPSCs		Yes					
	USP12	Nucleoplasm	Primary neurons		Yes	OE	↓			Aron et al. (2018)
						RNAi	↑			
						OE mutant	↓			
			HD patient iPSCs			OE	↓			
			Drosophila			OE	↓			
						RNAi	↑			
			HD patient line			OE	↓			
						OE mutant	↓			
	USP14	Plasma membrane, cytosol	PC6.3	changed localization		OE	↓	↓	↓ (Poly)	Hyrskyluoto et al. (2014)
						OE mutant		No effect		
			HeLa		Yes	OE		↓	↓ (Poly)	
						RNAi		No effect		
			Mouse—flHTT	no change						
	USP19	ER membrane, cytosol^¶^	HEK293			OE	↑	↑	↓ (Mono)	He et al. (2016)
						OE mutant	No effect	No effect	no effect	
						RNAi			↓ (Mono)	
			HEK293			OE		↑		He et al. (2017)

Table notes. All enzymes are expressed in brain tissue. Expression and localization data is obtained from The Human Protein Atlas (Uhlén et al. (2015), Sjöstedt et al. (2020), Thul et al. (2017)). If no localization data was available in The Human Protein Atlas, information was obtained from COMPARTMENTS (Binder et al. (2014)) and marked with ¶. Model*: If no HTT construct is mentioned, mHTT exon1 is expressed. flHTT = Full-length HTT. NLS = nuclear localization signal. OE = Overexpression. OE mutant = Overexpression ligase/catalytic dead mutant. OE mutant^1^ = Substrate binding mutant. OE mutant^2^ = Chaperone interaction mutant. Neg. OE^§^ = Dominant negative overexpression.

### 2.1 E1, E2 and E3 ubiquitinating enzymes

#### 2.1.1 UBA1

Ubiquitin-activating enzyme E1 (UBA1, also known as UBE1) is an E1 ubiquitin-activating enzyme and catalyzes the first step of the enzymatic ubiquitination cascade; namely activation of the ubiquitin molecules and subsequent transfer to E2 enzymes. Therefore, UBA1 plays a key role in the regulation of ubiquitin homeostasis, hence ubiquitination is involved in all downstream cellular processes, including protein degradation *via* the UPS. Nollen and colleagues performed a genome-wide RNA interference screen in *C. elegans* and identified UBA1 as one of the genes that increased polyQ-peptide aggregation upon knockdown (Nollen et al., 2004). Wade and colleagues studied UBA1 concerning the mHTT protein and found that E1 inhibition in brain lysate resulted in increased levels of oligomerized mHTT. Moreover, expression levels of endogenous UBA1 were decreased in striatum and cortex of HD mice, compared to cerebellum and periphery. In addition, UBA1 levels decreased with aging, suggesting that the decrease in enzyme levels contribute to the age-dependent onset of the disease (Wade et al., 2014). Despite the decrease in UBA1 levels during HD, an increase in ubiquitinated proteins was observed when brain lysate was incubated with mHTT. However, it is unclear whether this is a result of increased ubiquitination or decreased clearance (Wade et al., 2014). While UBA1 may play a role in polyQ-peptide and mHTT aggregation, it remains unclear whether UBA1 directly improves the turnover of HTT levels or whether the effect is indirectly mediated by other pathways, given the broad role of UBA1 in cellular pathways. The involvement of an E1 in many processes would limit UBA1 as a suitable target for HD treatment, as modulation of its activity would lead to too many side effects.

#### 2.1.2 HIP2

Ubiquitin-conjugating enzyme HTT Interaction Protein 2 (HIP2, also known as E2-25K/UBE2K) belongs to the class II E2 enzymes, which contain a Ubiquitin-Associated (UBA) domain C-terminal to the UBC domain. A UBA domain binds to ubiquitin, polyubiquitin chains, and ubiquitinated proteins. HIP2 can elongate K48-linked polyubiquitin chains and targets substrates for degradation (Chen and Pickart, 1990), yet the UBA domain of HIP2 preferentially binds to Lys63 polyubiquitin chains and facilitates the selective assembly of branched Lys48/Lys63 ubiquitin structures. In this way, HIP2 can target Lys63 polyubiquitinated substrates for proteasomal degradation *via* the formation of Lys63/Lys48-linked polyubiquitin signals (Pluska et al., 2021). HIP2 interacts with both wtHTT (Q16) and mHTT (Q44), as was shown with an *in vitro* binding assay (Kalchman et al., 1996). Colocalization studies suggest an interaction between HTT IBs and HIP2 in SH-SY5Y cells and post-mortem brain tissue, indicating that HIP2 colocalizes with HTT (de Pril et al., 2007). While knockdown of the HIP2 protein or deletion of its catalytic domain reduced mHTT IBs and polyQ-induced cell death in SH-Sy5Y cells, overexpression of HIP2 did not affect mHTT aggregation (de Pril et al., 2007), suggesting that HIP2 is not a limiting factor in controlling mHTT aggregation. Strikingly, knockdown of HIP2 in patient-derived induced pluripotent stem cells (iPSCs) had no effect on the number of mHTT IBs (Koyuncu et al., 2018). Since HIP2 does not colocalize with Q81-YFP IBs (Howard et al., 2007), this suggests that HIP2 requires the HTT protein sequence for interaction, not solely the polyQ tract that lacks lysine residues for ubiquitination. In line with this, silencing of HIP2 resulted in an increase in polyQ-peptide aggregation, yet the number of aggregates remained the same in HEK293 cells, and no change in polyQ solubility or overall levels was observed in a polyQ peptide *C. elegans* model (Howard et al., 2007). The discrepancy in colocalization between HIP2 and polyQ-peptides or mHTT suggests that HIP2 acts differently on polyQ-peptides and mHTT protein, and it needs to be confirmed whether HIP2 affects aggregate formation indirectly or can directly target mHTT and affect turnover.

#### 2.1.3 UBE2W

UBE2W is a class I E2 enzyme expressed in the nucleus. UBE2W generally does not transfer ubiquitin to free lysine residues, but instead prefers to specifically mono-ubiquitinate the N-terminal α-amino group of various proteins and specifically targets proteins with disordered N-termini independent of the amino acid sequence (Scaglione et al., 2013; Tatham et al., 2013; Vittal et al., 2015). At least 13 target proteins are known, most of which are targeted for proteasomal degradation by N-terminal ubiquitination *via* K48-linked polyubiquitin. Since the HTT N-terminus is also largely unstructured and disordered (Baias et al., 2017), Wang and colleagues tested the effect of UBE2W deficiency on the HD phenotype in different models. Overexpression of UBE2W in HEK cells increased aggregation, while overexpression of mutant UBE2W decreased aggregation by affecting mHTT solubility, suggesting that its catalytic activity affects mHTT aggregation directly. In line with this, UBE2W KO mouse primary cortical neurons showed reduced aggregation and decreased cell death (Wang et al., 2018), which may be the result of decreased toxic oligomer levels. While UBE2W knockout in a mHTT KI mouse model also led to a shift from oligomers to soluble HTT, it did not alter aggregation levels nor resulted in improved striatal functioning (Wang et al., 2018). Since no increase in ubiquitin levels was found in IBs and no changes in protein quality control pathways were observed in these mice, the mechanism by which UBE2W enhances mHTT solubility is still unclear. The authors suggest that UBE2W could promote aggregation by stabilizing HTT directly by ubiquitination or indirectly by increasing the probability of other post-translational modifications. UBE2W could also exert its effects by regulating other proteins that modify HTT, or the mechanism could be independent of ubiquitination (Wang et al., 2018).

#### 2.1.4 UBE3A

Ubiquitin ligase E3A (UBE3A), also known as E6AP, is a HECT domain E3 ubiquitin ligase with a zinc-binding fold called the AZUL (amino-terminal Zn-finger of UBE3A ligase) domain. UBE3A assembles K48-linked polyubiquitin chains (Wang and Pickart, 2005) and targets proteins, including polyQ proteins, for degradation (Mishra et al., 2008). UBE3A plays a role in synaptic plasticity (Maheshwari et al., 2012) and loss-of-function mutations in the *UBE3A* gene are associated with Angelman syndrome and Prader-Willi syndrome. In HD cell and mouse models, UBE3A interacts with both soluble and insoluble wtHTT and mHTT, but prefers to interact with longer polyQ-length repeats (Mishra et al., 2008; Bhat et al., 2014). In addition, UBE3A colocalizes with mHTT aggregates (Mishra et al., 2008; Mishra et al., 2009; Maheshwari et al., 2012; Bhat et al., 2014). While the levels of the UBE3A protein decrease in both WT and HD mice brain during aging, this decrease in nuclear UBE3A is more prominent in HD mice than in WT. Consequently, the interaction between UBE3A and the HTT protein is decreased in aged mice (Bhat et al., 2014). In contrast, *UBE3A* mRNA levels are increased in mHTT-expressing N2A cells (Mishra et al., 2008). Since the decrease in protein levels was observed in aging animals, this discrepancy could result from the lack of aging in immortalized cell lines. Alternatively, mRNA levels might not correlate with protein levels with UBE3A being decreased by post-transcriptional regulation (Mishra et al., 2008). Overexpression of UBE3A reduces mHTT aggregation (Bhat et al., 2014) and promotes mHTT degradation *via* the proteasome, in accordance with its role in K48-linked ubiquitination (Bhat et al., 2014; Maheshwari et al., 2014), and in parallel K63-linked ubiquitination was reduced (Bhat et al., 2014). Knockdown of UBE3A enhances aggregation (Mishra et al., 2008; Bhat et al., 2014; Maheshwari et al., 2014) and reduces ubiquitination of mHTT aggregates (Maheshwari et al., 2014). In addition, UBE3A knockdown accelerated the HD phenotype and reduced the life span in mice. In line with this, UBE3A overexpression reduced cell death in mHTT-expressing cells (Mishra et al., 2008). Taken together, UBE3A appears to be an important modulator of HD by improving HTT degradation by the proteasome, and maintaining sufficient levels of UBE3A during aging may modify mHTT levels and disease onset in HD patients.

#### 2.1.5 UBR5

UBR5 or EDD1/HHYD/KIAA0896/DD5 is a HECT domain E3 ubiquitin-ligase highly conserved in metazoans and has been implicated in a wide range of cellular processes, including DNA damage response, metabolism, transcription, and apoptosis. UBR5 contains UBA, UBR, and MLLE/PABC domains, with the UBA domain being used to interact with ubiquitin (Kozlov et al., 2007), the zinc finger Ubiquitin Recognin Box (UBR) domain being involved in N-end rule substrate recognition (Tasaki et al., 2005), and the MLLE/PACB domain (homologous to the C-terminal region of Poly-Adenylation Binding Protein) being used for protein-protein interactions and may regulate the transfer of ubiquitin to the substrate (Muńoz-Escobar et al., 2015). UBR5 has been identified as a possible mediator of the age of onset of HD in Genome Wide Association Studies (GWAS) (Lee et al., 2015). Patient-derived iPSCs show no mHTT aggregates or inclusion bodies being formed in time (Noormohammadi et al., 2016), suggesting that iPSCs exhibit an improved proteostasis regulation compared to other cell types. Interestingly, these iPSCs express increased levels of UBR5, which may contribute to the proposed improved proteostasis. Knockdown of UBR5 increased soluble HTT levels and triggered aggregate formation in mHTT-expressing iPSCs. Furthermore, knockdown of UBR5 in an invertebrate polyQ aggregation model also increased aggregation and neurotoxicity, while overexpression of UBR5 reduced aggregate formation in HD-cell models by inducing polyubiquitination and proteasomal degradation (Koyuncu et al., 2018). These results suggest an important role for UBR5 in the regulation of HTT turnover, and the increased ubiquitination of mHTT suggests that direct modifications by UBR5 are responsible for the improved degradation. Indeed, UBR5 is found to be involved in K11/K48-linked ubiquitination of mHTT (Yau et al., 2017). However, UBR5 can also regulate expression levels of UBE3A *via* polyubiquitination (Tomaić et al., 2011). Since UBE3A is associated with improved HTT degradation, as discussed above, UBR5 overexpression may counteract the beneficial effects of UBE3A.

#### 2.1.6 WWP1 and NEDD4

WWP1 (WW domain-containing E3 ubiquitin protein ligase 1) is a multifunctional HECT domain E3 ubiquitin-ligase and belongs to the NEDD4 subfamily. WWP1 contains an N-terminal C2 domain for membrane and protein binding, four tandem WW binding domains for substrate binding, and a C-terminal catalytic HECT domain for ubiquitin-protein ligase activity for both mono- and polyubiquitination (Courivaud et al., 2015). WWP1 predominantly binds to endosomes (Chen et al., 2008) and regulates various cellular biological processes, including transcription, protein transport, signal transduction, protein degradation, and viral budding. WWP1 has been implicated in aging and several diseases, such as cancers, infectious diseases, and neurological diseases (Zhi and Chen, 2012; Kuang et al., 2021). WWP1 protein levels were elevated in mice and N2a cells expressing mHTT, with WWP1 being recruited to aggregates. Overexpression of WWP1 in N2a cells resulted in increased soluble and insoluble mHTT levels and cell death, while downregulation enhanced cell viability and reduced mHTT levels. Furthermore, overexpression of WWP1 resulted in increased K63-ubiquitination of mHTT in cells, while downregulation decreased ubiquitination and improved degradation by the proteasome (Lin et al., 2016). Together these experiments show that WWP1 is involved in K63-linked ubiquitination and thereby inhibits proteasome-mediated HTT degradation, which depends on K48-linked ubiquitination. NEDD4 is an E3 ubiquitin ligase with the same yeast ortholog as WWP1 (Rsp5). Despite this, NEDD4 does not affect mHTT in the same manner as WWP1. While WWP1 increased soluble mHTT levels, NEDD4 did not alter soluble protein levels. Silencing of NEDD4 in HEK cells led to an increase in small aggregates (Peters et al., 2018). The effects of NEDD4 on total aggregation still need to be resolved.

#### 2.1.7 HACE1

HACE1 (HECT domain and Ankyrin repeat Containing E3 ubiquitin-protein ligase 1) is a HECT-type E3 that contains six Ankyrin repeats at the N-terminus and a C-terminal HECT domain. Ankyrin repeats are relatively common protein domains of ∼33 amino acids in length. These repeats act as scaffolds to facilitate specific protein-protein interactions through variable surface exposure (Mosavi et al., 2004). HACE1 is highly expressed in neuronal and glial cells and is involved in Golgi membrane fusion, the regulation of small GTPases, and protein degradation. Furthermore, HACE1 promotes the stability NRF2 (Da et al., 2021), which is an important regulator of the antioxidative stress response (Ma, 2013). Rotblat and colleagues found that HACE1 is reduced in the striatum of HD patients. However, this reduction was not observed in another highly affected region, the cortex. Expression of mHTT led to reduced *HACE1* mRNA in a mouse striatal cell line. HACE1 overexpression made mHTT-expressing striatal cells less sensitive to oxidative stress. This effect was independent of HACE1s E3 ligase activity but rather due to NRF2 induction (Rotblat et al., 2014). NRF2 was previously marked to be involved in mHTT degradation (van Roon-Mom et al., 2008). YAC128 HACE1 knockout mice show an impaired anti-oxidative stress response. Moreover, these mice present with aggravated motor and psychiatric deficits (Ehrnhoefer et al., 2018). However, it must be noted that, although not statistically significant, HACE1 knockout leads to phenotypic impairments. It can, therefore, not be excluded that the aggravated phenotype is a result of the accumulation of the different impairments caused by mHTT expression and HACE1 knockout. Taken together, HACE1 levels decrease in the striatum during HD and due to its role in protecting against HTT-induced oxidative stress, the decrease in HACE1 levels could be responsible for the increased pathology in the striatum.

#### 2.1.8 TRAF6

TNF receptor-Associated Factor 6 (TRAF6) is a RING E3 ligase and member of the TRAF protein family and contains an N-terminal RING domain, four zinc finger (ZF) motifs, and a C-terminal TRAF domain. The N-terminal RING and ZF1 domains are used for K63 polyubiquitination (Yin et al., 2009)*.* The TRAF domain consists of two distinct subdomains: the TRAF N-domain, which is a coiled-coil domain, and a highly conserved TRAF-C domain (Yin et al., 2009). The TRAF-C domain is responsible for protein-protein interactions (H. Ye et al., 2002). TRAF6 promotes polyubiquitination of its substrates (Yin et al., 2009), and is not limited to specific linkage types. It has been shown to promote K63-polyubiquitination (Geetha et al., 2005; Linares et al., 2013), K48/K63 polyubiquitination of ATG9A (Wang et al., 2022), as well as a-typical polyubiquitination *via* K6-, K27, and K29-linkages of proteins involved in Parkinson’s disease (Zucchelli et al., 2010). TRAF6 interacts with wtHTT and both soluble and aggregated mHTT in HEK cells, mice- and human cortex. In addition, *TRAF6* mRNA and protein levels were increased in post-mortem cortex tissue of HD patients (Zucchelli et al., 2011). Overexpression of TRAF6 in HEK cells led to increased aggregation number and size but did not alter total HTT levels. Moreover, overexpression did not affect wtHTT localization. TRAF6 enhanced the ubiquitination of wtHTT and mHTT with atypical ubiquitin chains linked at K6, K27, and K29 (Zucchelli et al., 2011). Despite the role of TRAF6 may not be specific for mHTT, further research needs to be performed on the role of this aberrant ubiquitination in HTT clearance.

#### 2.1.9 PJA1

PJA1 or PRAJA1/RNF70 is a RING ubiquitin ligase with yet unknown function. It is expressed in various tissues with the highest expression levels found in brain and has been associated with neurodevelopmental disorders, such as epilepsy and/or craniofacial abnormalities (Suzuki et al., 2020). The expression of both wtHTT and mHTT leads to decreased mRNA and protein levels of PJA1. While PJA1 interacts and colocalizes with wtHTT and mHTT in cell models (Ghosh et al., 2021; Watabe et al., 2022) aggregates in post-mortem brain samples of HD patients stained negative for PJA1 (Watabe et al., 2022). Overexpression of PJA1 reduced mHTT aggregation and soluble levels of wtHTT (Ghosh et al., 2021; Watabe et al., 2022). Silencing of PJA1, on the other hand, increased both soluble and aggregated mHTT (Ghosh et al., 2018). Although not tested for HD, PJA1 lowers toxicity in a yeast and *Drosophila* SCA3 model, another polyQ aggregation disorder. To conclude, there seems to be a link between PJA1 and HTT, irrespective of the polyQ repeat length. However, the exact role in HD and the affected mechanism remains to be resolved.

#### 2.1.10 UHRF2

Recently it was shown that cytoplasmic and nuclear aggregates of mHTT have a different biochemical composition, as well as different interactome and structural properties, which suggests that different mechanisms drive aggregate formation, clearance, and toxicity, depending on the subcellular localization (Riguet et al., 2021). When the Protein Quality Control (PQC) system was studied in the nucleus, overexpression of yeast nuclear E3 ligase San1p in yeast and HeLa cells increased cell viability and reduced aggregation of nuclear expressed mHTT by decreasing its half-life. Additionally, total protein levels of wtHTT were also lowered by San1p overexpression (Iwata et al., 2009). Previously it was already found that San1p mediated the ubiquitination of aberrant nuclear proteins leading to their proteasomal degradation. San1p only targeted the mutant aberrant proteins, not the wild-type counterparts, suggesting that San1p is part of a protein quality control system in the nucleus (Gardner et al., 2005). The E3 ubiquitin ligase Ubiquitin Like with PHD and RING Finger Domains 2 (UHRF2) is the closest mammalian homolog of San1p and is a nuclear protein involved in DNA methylation, histone modifications, cell cycle regulation, and DNA repair, and can target proteins for degradation by the proteasome. UHRF2 contains an N-terminal ubiquitin-like domain (UBL) domain, a Tandem-Tudor domain (TTD) and Plant Homeodomain (PHD) for the recognition of histone modifications, a SET and RING associated domain (SRA) for DNA binding, and a RING domain for E3 ubiquitin ligase activity. Similar to San1p, UHRF2 colocalizes with nuclear mHTT aggregates in mammalian cells and R6/2 mice. In addition, overexpression of UHRF2 in cells reduced neurotoxicity and cell death, and reduced nuclear aggregation by decreasing mHTT and wtHTT half-life. Furthermore, increased ubiquitination of mHTT was found after UHRF2 overexpression (Iwata et al., 2009). This data suggests that UHRF2 is involved in HTT turnover, irrespective of its polyQ repeat length, by promoting its ubiquitination. The route of degradation is not investigated.

#### 2.1.11 HRD1

HRD1/Der1 is an ER membrane-spanning E3 ubiquitin ligase that contains five transmembrane domains and a RING finger domain that is facing the cytoplasm, and is induced by the unfolded protein response pathway (UPR) and mediates the degradation of misfolded proteins in ER-associated protein degradation (ERAD). In ERAD, HRD1 functions as a pore and can regulate the transport of misfolded proteins from the ER lumen to the cytoplasm *via* ubiquitination. Auto-ubiquitination of the HRD1 RING domain generates a high-affinity substrate binding site on the cytoplasmic side of HRD1, where it ubiquitinates these substrates (Bordallo et al., 1998; Vasic et al., 2020). Next, p97/VCP pulls the substrate out of the membrane and hence the substrate can be degraded by the proteasome. Cells overexpressing mHTT exhibit increased levels of HRD1 protein, but not mRNA (Yang et al., 2007), suggesting post-transcriptional regulation of protein levels. By immunoprecipitation, it was shown that HRD1 interacts with both wtHTT and mHTT. Moreover, microscopy assays show that HRD1 recruits HTT to the ER. While HRD1 knockdown increased soluble mHTT levels, overexpression decreased mHTT aggregates and soluble levels, in a polyQ-length dependent manner. The decrease in mHTT half-life depended on the HRD1 RING domain and the ATPase VCP/p97 (Yang et al., 2007). Although HRD1 interacts with both wtHTT and mHTT, improved ubiquitination was only observed in HTT with high polyQ repeat lengths (Yang et al., 2007). HRD1 improved HTT degradation *via* improved ubiquitination and protected against mHTT-induced cell death, but the exact mechanism needs further study.

#### 2.1.12 Parkin

Parkin (PRKN) is a RING-BETWEEN-RING (RBR) E3 ligase that is located in the cytoplasm. It contains an N-terminal UBL domain and four zinc-coordinating RING-like domains: RING0, RING1, IBR, and RING2. The UBL domain controls cellular PRKN levels and activity in addition to binding to SH3-, ubiquitin interacting motif (IUM) domains and the proteasome complex (Finney et al., 2003; Sakata et al., 2003; Trempe et al., 2009; Chaugule et al., 2011). Polyubiquitin chains formed by PRKN include K6, K11, K48, and K63 linkages (Ordureau et al., 2014). PRKN is upregulated during the UPR and plays a role in the degradation of misfolded ER proteins *via* the ERAD pathway (Imai et al., 2000). It interacts with HSP70 and ubiquitin ligase CHIP, and in turn, CHIP positively regulates the E3 ligase activity of PRKN (Imai et al., 2002). The E3 activity of PRKN is generally inhibited but is activated by recruitment to the mitochondrial outer membrane (MOM) in response to mitochondrial depolarization (Narendra et al., 2008; Matsuda et al., 2010). Activated PRKN ubiquitinates primarily MOM proteins and promotes their degradation *via* the UPS (Chan et al., 2011). Increased mRNA and protein levels of PRKN were observed in juvenile HD fibroblasts (Q68 and Q86) (Aladdin et al., 2019), and PRKN colocalizes with mHTT inclusion bodies in the brain of mHTT-Q72 YAC72 mice and with both cytoplasmic and nuclear inclusions in human brain. Co-immunoprecipitation also showed an interaction between PRKN and mHTT, but not with wtHTT, suggesting a preference for PRKN for polyQ-expanded HTT (Tsai et al., 2003). When the HD R6/1 mouse model was crossed with PRKN null mice to study the effect of partial suppression of PRKN on HD, a decreased number of aggregates in the striatum of R6/1/PK^+/−^ mice was observed, while the effect was less severe or absent in other brain regions. Despite the reduction in aggregation, increased striatal cell death and exacerbated motor and behavioral deficits were observed (Rubio et al., 2009). This could indicate that mHTT moves from an aggregation state towards a state with more toxic polymers after PRKN knockdown. Since PRKN knockdown leads to increased LC3II/LC3I ratios, it was suggested that the autophagy pathway becomes more active and may be responsible for the decreased aggregation (Rubio et al., 2009).

#### 2.1.13 HOIP and OTULIN

The E3 ubiquitin ligase HOIP belongs to the RBR family and is the only known E3 ligase that can assemble linear M1-linked polyubiquitin chains and functions as part of the LUBAC complex (Kirisako et al., 2006). HOIP uses its RING2 domain and a newly identified Linear ubiquitin chain Determining Domain (LDD) in its C-terminus to assemble linear ubiquitin chains (Smit et al., 2012). LUBAC assembles linear ubiquitin chains, preferably on pre-existing K63-linked polyubiquitin chains and possibly on other polyubiquitin linkage types (Emmerich et al., 2013). Linear polyubiquitin plays a role in various cellular processes, including immune signaling, defense against intracellular pathogens, protein quality control, and cell death regulation (Iwai et al., 2014; Noad et al., 2017; Well et al., 2019). Aggregates and oligomeric aggregate precursors of mHTT were found to be ubiquitinated with linear ubiquitin and contained several components of the LUBAC complex, including HOIP, HOIL-1L, and SHARPIN (Well et al., 2019). HOIP was recruited to mHTT by p97/VCP, resulting in linear polyubiquitin formation covering the interactive surface on mHTT. Silencing of HOIP reduced mHTT linear ubiquitination and increased the interaction of mHTT with transcription factor Sp1, which resulted in transcriptional dysregulation of LUBAC complex components. HOIP silencing also increased aggregation and proteotoxicity. These effects were counteracted by silencing OTULIN, which is the only described mammalian DUB that specifically hydrolyzes linear polyubiquitin (Keusekotten et al., 2013). Linear ubiquitination also facilitated proteasome-dependent degradation of mHTT (Well et al., 2019). Linear polyubiquitination thus seems to have a protective effect in HD where it decreases the misfolded protein’s toxic potential and stimulates the removal *via* the proteasome. Since linear ubiquitination is a very specific process, it might be a promising target for drug development for HD and other protein aggregation-based diseases (Well et al., 2019).

#### 2.1.14 CHIP

The C-terminus of HSP70-interacting protein (CHIP) is a U-box ubiquitin ligase (UULs) that contains a U-box domain structurally related to the RING finger domain. CHIP is an E3 ubiquitin ligase that links the protein folding machinery with the UPS, interacts with molecular chaperones HSP70, HSC70, and HSP90 *via* three tetratrico peptide repeat (TPR) domains, and interacts with the proteasome *via* its E4/U-box domain. Thus, CHIP possibly acts as a regulator in triage decisions; it sends the substrate for refolding or proteasome-dependent degradation. CHIP uses its TRP domain, not the E4/U-box domain, to reduce aggregation of polyQ-GFP and GFP-tagged N-terminal mHTT in transfected cell lines, suggesting that polyQ proteins are refolded through the action of CHIP in order to keep them in a soluble state and hence reduce aggregation (Miller et al., 2005). CHIP levels increased after mHTT expression in astrocytes but not in neurons, which suggests cell type-specific regulation (Zhao et al., 2017). Interestingly, no interaction or colocalization was found in neurons or a mouse brain region high in neuronal cell content. However, CHIP did interact or colocalize with mHTT in astrocytes and brain regions high in astrocyte abundance (Zhao et al., 2017). Colocalization and interaction of mHTT and CHIP were also observed in N2a cells (Jana et al., 2005). Studies on CHIP overexpression in cellular models of HD showed a reduction of mHTT aggregation and increased soluble levels, which was more prominent when HSC70 was present (Miller et al., 2005). In addition, CHIP overexpression reduced polyQ-induced pathology in cells, zebrafish, and *Drosophila* (Miller et al., 2005; Al-Ramahi et al., 2006). In line with these results, HD mice that were haploinsufficient for CHIP displayed an accelerated disease phenotype (Miller et al., 2005). While overexpression of a ligase-dead mutant still improved mHTT solubility, overexpression of a chaperone-dead mutant was unable to induce reduced aggregation of a polyQ-expanded protein (Miller et al., 2005). In addition, they found no alterations in mHTT ubiquitination after CHIP knockdown in an HD mouse model or improved mHTT degradation rate after overexpression in cells. This indicates that the chaperone function of CHIP is responsible for the shift in solubility but not mHTT turnover. It is important to note that the role of the ligase-dead mutant was excluded based on only a polyQ-peptide construct and not on the mHTT protein. Al-Ramahi and colleagues found that CHIP could not suppress the toxicity of a bare 127Q stretch but could efficiently suppress the toxicity of an N-terminal mHTT fragment in a *Drosophila* model for HD (Al-Ramahi et al., 2006). Their conclusion is that the protein context is important for the action of CHIP, maybe due to the presence of lysine residues that can be ubiquitinated. It is worth mentioning that the constructs used for polyQ proteins in the study of Miller and colleagues contain GFP, therefore probably offering a suitable protein context for CHIP. Zhao and colleagues observed decreased K48-linked ubiquitination of mHTT after CHIP downregulation in mouse brain (Zhao et al., 2017). In addition, Jana and colleagues found an increase in ubiquitination of mHTT after CHIP overexpression and found that CHIP ligase mutant could not lower aggregation and accompanied cell death (Jana et al., 2005). The authors conclude that the decreased aggregation is mediated by proteasomal degradation. However, they do not measure mHTT soluble levels or turnover, as this was only tested for ataxin 3, and a proper control condition measuring the sole effect of proteasome inhibition is missing. To conclude, CHIP overexpression decreases aggregation and alleviates pathology, however, further research needs to be performed to draw conclusions on the involved mechanism.

#### 2.1.15 SCF ubiquitin-ligase complex components CUL1, SKP1 and FBXW7

Skp, Cullin, F-box containing complex (or SCF complex) is a multiprotein E3 ubiquitin ligase complex that belongs to the cullin family of RING-finger E3s. The SCF complex consists of three core subunits (the adaptor protein SKP1, a major structural scaffold protein CUL1, an RBX1 RING-finger E3 ligase domain), and a variable F-box protein. Independent of the SCF complex, the F-box protein recognizes and interacts with specific target proteins. Substrate recognition often depends on post-translational modifications, and each F-box protein may recognize several different substrates. Next, the F-box protein binds to the adaptor protein SKP1 that links the F-box protein to CUL1, thereby recruiting the substrate to the E2 enzyme and the RBX1 domain. RBX1 contains a RING-finger domain to which the E2 enzyme binds. Different Cullins and F-Box Proteins combinations can generate a wide variety of E3 ubiquitin ligases targeting different substrates. The SCF complex targets proteins with mono- or polyubiquitin chains and can regulate various molecular processes, including protein localization, protein activity, or protein targeting for degradation, of which the latter one is best studied. In this case, the SCF complex is involved in the polyubiquitination of target proteins. Bhutani and colleagues found that the levels of CUL1 and SKP1 were reduced in HD mice brain. In addition, lower CUL1 levels were observed in a cellular model and a *Drosophila* model of HD. Expression of a dominant negative mutant of CUL1 resulted in increased aggregation in cell culture. The silencing of CUL1 or SKP1 in *Drosophila* resulted in increased aggregation and toxicity. Since the combination of mHTT expression and CUL1 or SKP1 silencing was lethal, results were based on the few surviving animals (Bhutani et al., 2012). Gomez-Pastor and colleagues found increased levels of the F-box protein FBXW7 and reduced levels of the stress-protective transcription factor HSF1 in HD models. FBXW7 is an F-box protein of the SCF complex that controls the proteasome-mediated degradation of various oncoproteins. Restoration of HSF1 levels prevented mHTT aggregation. Further research is necessary to understand how mHTT expression drives enhanced expression levels of FBXW7 (Gomez-Pastor et al., 2017). The proposed pathway explaining the effect of FBXW7 on mHTT through HSP1 regulation could also account for the described effects of CUL1/SKP1.

### 2.2 DUBs

#### 2.2.1 Ataxin 3

Ataxin 3 (ATXN3) belongs to the Josephin family of deubiquitinating enzymes and contains an N-terminal Josephin domain (JD) with a papain-like fold, while the C-terminus is unstructured and contains two or three UIMs and a polyQ stretch (Masino et al., 2003). The UIM motif binds both K48- and K63-linked polyubiquitin chains but prefers to cleave K63-linkages, especially when present in K48- and K63-mixed polyubiquitin chains (Winborn et al., 2008). The JD trims polyubiquitin chains down to four residues (Burnett et al., 2003), while it has weak or no activity against shorter chains. ATXN3 is involved in various biological processes such as transcription, protein homeostasis, cytoskeleton regulation, and degradation of misfolded chaperone substrates. ATXN3 can prevent self-ubiquitination of PRKN, thereby modulating PRKN activity (Durcan et al., 2012). When null mice for ATXN3 were crossed with an HD knock-in mouse model, it was observed that ATXN3 did not contribute significantly to HD progression. Decreased levels of ATXN3 only mildly aggravated age-dependent motor deficits, but no alterations were observed with respect to inclusion body formation, ubiquitination of inclusion bodies, or levels of the mHTT or wtHTT protein (Zeng et al., 2013). ATXN3 was shown to be sequestered in mHTT aggregates in HD brain, and wtHTT was shown to interact with ATXN3 in cell models, together with other subunits of the transcription-coupled DNA repair complex. This complex recognizes DNA damage and mediates DNA repair during transcription elongation. Expression of mHTT decreased the activity of ATXN3, which negatively affected transcription and DNA repair and might trigger neurotoxicity in HD (Gao et al., 2019).

#### 2.2.2 YOD1

YOD1 is a conserved deubiquitinase belonging to the OTU family (Ernst et al., 2009) and prefers cleaving K48- and K63-linked polyubiquitin (Tanji et al., 2018). By associating with p97, it facilitates protein dislocation from the ER (Ernst et al., 2009) and proteasomal degradation of ERAD model substrates (Sasset et al., 2015). YOD1 protein levels were increased in mHTT-expressing cells (Tanji et al., 2018). Overexpression of YOD1 decreased mHTT levels and mHTT-induced cell death. These effects were not observed with catalytically inactive YOD1, indicating it depends on its deubiquitinase activity. YOD1 also altered proteasomal activity. While mHTT induced trypsin and chymotrypsin activity in cell lysate, this increased activity was absent in the presence of YOD1 (Tanji et al., 2018).

#### 2.2.3 USP7

USP7 or HAUSP belongs to the Ubiquitin Specific Protease (USP) family of deubiquitinating enzymes and is involved in various biological pathways, including apoptosis, transcription regulation, DNA replication, and neuronal development. It contains an N-terminal polyQ region, followed by a TRAF domain, a catalytic domain and a C-terminal UBL domain. USP7 has many different interactors, which can function as scaffolds, activity modulators, or substrates for deubiquitination. Well-known substrates for USP7-mediated deubiquitination include the tumor suppressor protein p53 and WASH, a protein that is essential for endosomal protein recycling. USP7 regulation and complex formation is reviewed elsewhere (Kim and Sixma, 2017). USP7 interacts with full-length wtHTT and mHTT in the striatum and frontal cortex of a knock-in mouse model (zQ175) of HD, but USP7 prefers interacting with the mHTT protein. A proximity ligation assay in iPSC cells derived from an HD patient confirmed the interaction between USP7 and HTT in a human model. The data suggest that USP7 preferentially interacts with polyQ-expanded proteins (Pluciennik et al., 2021). It remains to be resolved whether USP7 is involved in the direct de-ubiquitination of HTT. Also, the role of the wtHTT and mHTT interaction with USP7 is still unclear.

#### 2.2.4 USP12

While the role of USP12 is not well established, several substrates for USP12 are identified, including histone H2A/H2B, Notch, MDM2, and androgen receptor (Joo et al., 2011; Moretti et al., 2012; Burska et al., 2013; McClurg et al., 2018). USP12 colocalizes with mHTT IBs in primary neurons. Overexpression of USP12 increases cell survival after mHTT expression in primary neurons and HD patient cells and decreases the mHTT-induced phenotype in *Drosophila*. Surprisingly, overexpression of a catalytic-dead mutant resulted in the same protective effects. This indicates that the role of USP12 in HD pathology is independent of its deubiquitinase activity. In addition, no change in mHTT half-life was detected, which implies that USP12 is not involved in mHTT turnover. USP12 does lead to autophagy induction, which is needed for the neuroprotective effects of USP12 in HD. The authors suggest that autophagy could be involved by degrading a subpopulation of mHTT that is not measured in their half-life assay. It could also be that autophagy regulates HD pathology by degrading other substrates, and by improving general proteostasis, mHTT-induced toxicity is reduced (Aron et al., 2018).

#### 2.2.5 USP14

The deubiquitinating enzyme USP14 belongs to the USP family and contains N-terminal UBL and C-terminal USP domains. The UBL domain is an important regulator of proteasomal activity, and the USP domain is required for its deubiquitinating activity. USP14 reversibly associates with the 19 S cap of the proteasome. It is involved in the regulation of deubiquitination of proteasome substrates, hence is involved in editing and rejecting substrates for proteasome-dependent degradation. Rapid deubiquitination can reduce the dwell time at the proteasome and prevent substrate degradation. Deubiquitination at the proteasome also enables the recycling of ubiquitin molecules. USP14 can remove single ubiquitin moieties from the tip of polyubiquitin chains (Hu et al., 2005), but it can also remove multiple polyubiquitin chains from a substrate *en bloc* until a single chain remains (Lee et al., 2016). In summary, the specificity of the proteasome can be regulated by deubiquitination. USP14 interacts with wtHTT and mHTT, as shown by co-immunoprecipitation experiments in cell lysates (Hyrskyluoto et al., 2014). While no change in USP14 total levels was found in BACHD mice and HTT-expressing cells, the localization of USP14 changed from the ER membrane fraction to the cytosolic fraction after mHTT expression. Overexpression of wild-type, but not catalytic inactive, USP14 in cells resulted in reduced aggregation and cell death. The decrease in the aggregation after USP14 expression was blocked by proteasome inhibition, while an autophagy inhibitor did not alter mHTT aggregation. This implies that the proteasome mediates the decrease in aggregates. However, USP14 mainly affected insoluble mHTT levels, not soluble mHTT levels (Hyrskyluoto et al., 2014), suggesting that the effect of USP14 is not mediated by directly improving the degradation of mHTT. USP14 inhibits mHTT-induced caspase-3 activity, which is involved in the regulation of cell death, and mHTT-induced activation of IRE1α, a transmembrane protein in the ER that acts as an ER stress sensor, in cells and BACHD mice (Hyrskyluoto et al., 2014). Although USP14 affects mHTT aggregation, the positive effects on cell viability could also be mediated by caspase-3 or IRE1α.

#### 2.2.6 USP19

USP19 belongs to the USP family of deubiquitinating enzymes. It contains two CHORD-SGT1 domains at the N-terminus, potentially used to interact with the HSP90 chaperone (Zhang et al., 2008). Furthermore, it has a central USP domain with a UBL domain and a MYND zinc finger. Alternative splicing results in the generation of two major isoforms that differ in their C-termini. For instance, one isoform contains a transmembrane domain that anchors USP19 to the endoplasmatic reticulum and is involved in ERAD. The other isoform contains a relatively hydrophilic region and an EEVD motif in the C-terminus and has a cytoplasmic localization. USP19 plays a role in the protein quality control system, protein homeostasis, muscle development, tumorigenesis and controls the half-life of several proteins (Rossi and Rossi, 2022). Overexpression of cytoplasmic localized isoform of USP19 in mHTT-expressing cells increased mHTT levels and aggregation. Overexpression of a catalytic-dead mutant was not able to produce this increase. In addition, USP19 overexpression increased mHTT-induced cell death. Silencing USP19 showed the opposite effects and decreased soluble mHTT levels. USP19 may work together with the chaperone HSP90, which recognizes unfolded mHTT, thereby playing a key role in triage decisions for disease-related polyQ-expanded substrates (He et al., 2016; He et al., 2017).

## 3 Discussion

**FIGURE 2:**
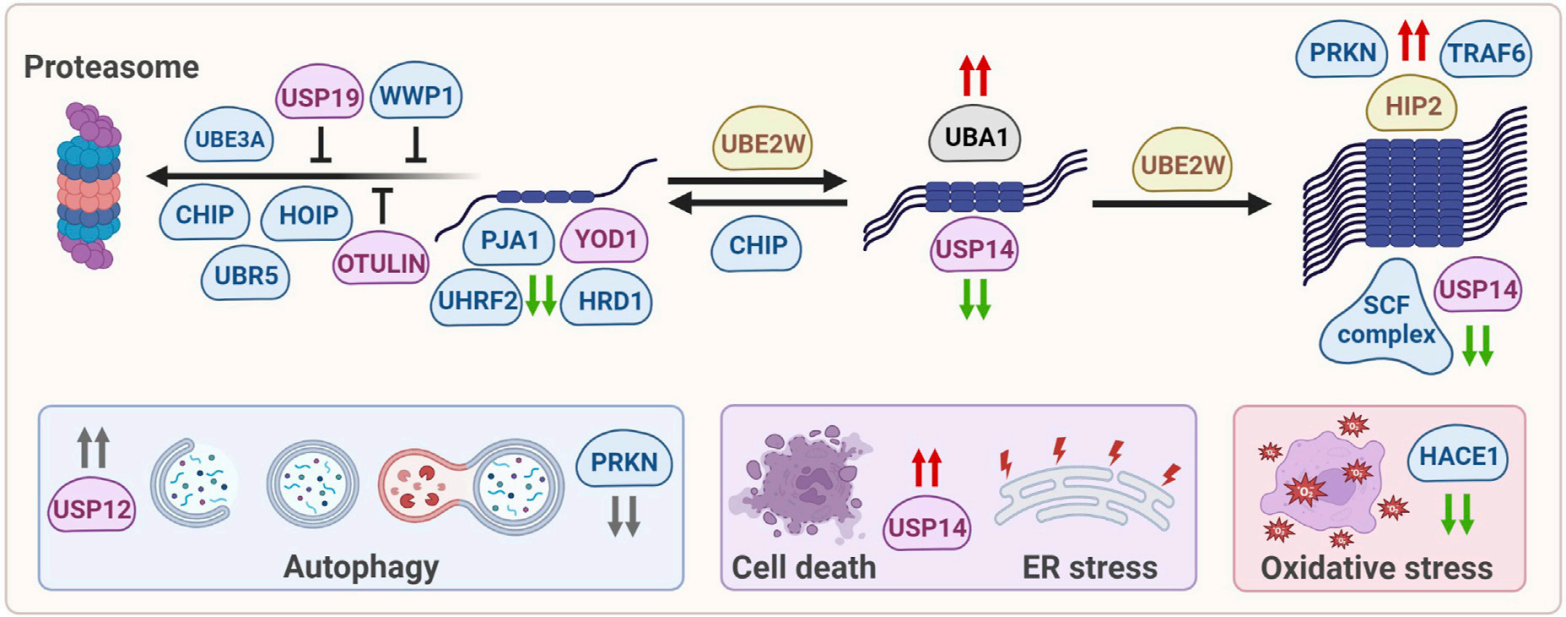
Ubiquitin-modifying enzymes that affect mHTT protein levels, solubilization, oligomerization, aggregation, degradation and mHTT-induced cellular stress. E1 enzymes are shown in grey, E2 enzymes are shown in yellow, E3 enzymes are shown in blue, DUBs are shown in pink. Green arrows: enzymes reduce mHTT levels, red arrows: enzymes increase mHTT levels, grey arrows: enzymes affect a process but not mHTT levels.

The various ubiquitin-modifying enzymes that directly or indirectly affect mHTT aggregation, as described in this review, affect mHTT at various levels, ranging from soluble, monomeric mHTT levels or alter oligomerization and aggregation of mHTT. They can also be involved in targeting mHTT towards the proteasome, or modifying autophagy in HD models, although there might be no direct link with the degradation of mHTT *via* this pathway. Indirect effects include regulation of ER stress, cell viability and oxidative stress in HD (Figure 2).

### 3.1 Effects of ubiquitin-modifying enzymes on mHTT levels and HD pathology

#### 3.1.1 Ubiquitin-modifying enzymes that affect mHTT aggregation and facilitate degradation by the proteasome

Several ubiquitin ligases improve soluble wtHTT and/or mHTT degradation by the proteasome *via* poly-ubiquitination, including the E3 ubiquitin ligase UBE3A, UBR5 and HOIP, as well as the U-box ubiquitin ligase and CHIP. Preventing the reported age-related decrease in some of these enzymes would be a therapeutic strategy for HD, for example by enhancing the activity of the remaining pool of UBE3A, or increasing their expression level. Similarly, inhibiting the activity or decreasing the expression levels of particular DUBs including OTULIN and USP19, but also the CHIP inhibiting protein HspBP1 or the ligase WWP1, might be a strategy to reduce or prevent HD progression by improving proteasomal turnover. In addition, there are several ubiquitin-modifying enzymes affecting soluble wtHTT and/or mHTT protein levels, but their mechanism of action remains to be resolved, including the E3 ubiquitin ligase PJA1 and UHRF2. Yet they also affect wtHTT levels, which is not a preferable strategy for treating HD. In contrast, the ligase HRD1 affects soluble and aggregated mHTT levels by interacting with the VCP/p97 complex (Yang et al., 2007) that functions as a disaggregase of misfolded and polyubiquitinated proteins, including mHTT, which may facilitate mHTT accessibility for degradation. Finally, proteasomal degradation of mHTT might be facilitated by enzymes that affect mHTT solubilization. Here, the nuclear E2 enzyme UBE2W and E3 ubiquitin ligase CHIP were found to modify the solubilization of mHTT. However, improving solubilization may also induce mHTT-induced toxicity when not accompanied by improved ubiquitination and subsequent degradation, as shown when UBE2W levels were reduced (Wang et al., 2018). This is different for the ligase CHIP that can improve solubilization of mHTT by chaperone-mediated refolding, as well as proteasome-dependent degradation, thereby reducing pathology (Miller et al., 2005; Al-Ramahi et al., 2006). Therefore, activation of CHIP might be a possible target for drug development, as well as inhibition of HspBP1 as mentioned above. Last but not least is the deubiquitinating enzyme USP14 which targets oligomerized mHTT and subsequently improves proteasome-dependent degradation (Hyrskyluoto et al., 2014), suggesting that even oligomerized mHTT can still become a proteasomal target.

Increased expression of various enzymes described above affect mHTT aggregation and toxicity, including CUL1, SKP1, TRAF6 and PRKN, but since their mechanism-of-action is unknown they are for now no strategic targets for therapy. Other enzymes are known to improve autophagy, as was shown for the deubiquitinating enzyme USP12 that induces neuronal autophagy. Yet while USP12 can rescue mHTT-mediated neurodegeneration in different HD models *via* induction of autophagy, mHTT IB formation was not affected, suggesting that neuroprotection is obtained by improving clearance of other proteins than mHTT (Aron et al., 2018). In contrast, partial suppression of PRKN improved autophagy flux and reduced mHTT aggregation in HD mice, yet increased neuronal cell death and exacerbated motor and behavioral deficits (Rubio et al., 2009), suggesting that keeping mHTT in a more oligomeric species does not automatically result in improved recognition and clearance by autophagy.

#### 3.1.2 Ubiquitin-modifying enzymes that affect cellular stress and cell death in HD

Reducing oxidative and ER-stress indirectly contributes to HD-related cell stress, and both HACE1 and USP14 play a role in these processes, respectively. Overexpression of HACE1 reduces oxidative stress *via* NRF2 induction while HACE1 knockout mice show an impaired anti-oxidative stress response and aggravated motor and psychiatric deficits (Ehrnhoefer et al., 2018). The deubiquitinating enzyme USP14 lowers both mHTT aggregation and mHTT-induced cell death, which is mediated by the reduced interaction between USP14 and IRE1α in the ER membrane in mHTT-expressing cells and BACHD mice, thereby reducing ER-stress *via* IRE1α.

### 3.2 Comparing HD studies

While numerous enzymes in all stages of the ubiquitination pathway (E1s, E2s, E3s and DUBs) have been identified to influence HTT levels or the HD phenotype (Figure 2) and reviewed in their corresponding sections above, some aspects of the study design require attention when comparing different studies. Studies on the same enzyme can use different versions of the HTT protein, including the full-length protein, but also different truncated N-terminal HTT fragments. Especially shorter N-terminal mHTT fragments are aggregation-prone, while the full-length mHTT protein is mostly soluble. Often HTT fragments are expressed with a tag, which can significantly influence a protein’s localization, interaction with other proteins and stability. It is preferred to use untagged HTT, to exclude the interference of the used tag on the protein’s behavior. In addition, different polyQ-lengths can be used, and differences in length affect the folding, post-translational modifications, protein-protein interaction, aggregation and toxicity. Some studies use polyQ-peptides instead of mHTT peptides and thus lack the HTT sequence. These polyQ-peptides show aggregation properties, but these peptides do not have the mHTT protein context, which can affect protein-protein interactions. All these differences can cause different effects in HD models.

In addition, different methods to analyze soluble and insoluble HTT protein levels are used in different studies. The mHTT protein can be present in different forms ranging from soluble monomers and High Molecular Weight (HMW) polymers to insoluble mHTT aggregates and IBs, and different methods can be used to study these different forms of mHTT. Many studies use fluorescently tagged HTT to quantify the number of IBs by microscopy, including determination of aggregate size, and this is different from biochemical assays using protein lysates. Here, lysate fractions can be divided into Triton-X100 insoluble aggregates, Triton-X100 soluble monomers, and HMW polymers, which can be detected with SDS-PAGE western blot, and it is important to quantify both HTT aggregates and soluble levels to get a complete picture of the effects on total protein levels. Is an intervention responsible for a shift between the soluble and insoluble fraction, or does it affect total protein levels *via* the proteostasis machinery?

Finally, the role of enzymes acting in the ubiquitination pathway can be investigated in many different models, ranging from *in vitro* experiments using isolated enzymes to *in vivo* experiments using small animal models. In cells, the experimental conditions are easily controllable, and it is relatively easy to manipulate enzyme levels. *In vivo* models, on the other hand, offer the potential to study the effects of enzyme modulation in an entire system and to study the effects on HD pathology. HD pathology only manifests later in life, indicating that aging processes are important in the etiology of the disease. These aging processes are not always present in immortalized cell lines, which could contribute to differences in experimental outcomes. This ‘young’ phenotype is especially present in iPSCs. The fact that most iPSC do not show aggregation already suggests a different proteostasis network, as suggested by Koyuncu et al. (2018). In addition, the brain consists of many cell types, which do not all show the same pathological features. Indeed, Zhao and colleagues showed differences in proteasome-dependent degradation of mHTT *via* CHIP between astrocytes and neurons, as CHIP activity was inhibited in neurons but not in astrocytes (Zhao et al., 2017). Many enzymes are only described in one or few studies or research groups, while the evidence would be stronger when more independent research groups repeat experiments.

### 3.3 Conclusion

Ubiquitination is an important process to target proteins for degradation through the UPS or autophagy. Not surprisingly, several enzymes involved in ubiquitination are found to be linked with HD. The levels of many of these enzymes are changed in HD compared to healthy conditions, which suggests a dysregulation of ubiquitin-regulated pathways, including proteostasis. Modulating the ubiquitination system components as therapeutic targets has gained more attention (Cohen and Tcherpakov, 2010). However, clinical agents or small molecule inhibitors have only been developed for a small fraction of these enzymes. A cell-permeable inhibitor for the E1 enzyme UBA1 is developed and used in clinical trials, but given the pleiotropic behavior of E1 enzymes, these inhibitors could only be used in acute settings such as aggressive cancers (Hyer et al., 2018). E3 ubiquitin ligases confer a high substrate specificity and would therefore be attractive targets for drug development. However, the lack of apparent druggable sites in E3 enzymes makes this challenging. On the other hand, due to their well-defined catalytic clefts, DUBs are appealing as prospective therapeutic targets (Cohen and Tcherpakov, 2010). Interestingly, mHTT is ubiquitinated at K6 and K9, while wtHTT is not (Sap et al., 2019; Hakim-Eshed et al., 2020), and the differences in ubiquitination as well as phosphorylation between wtHTT and mHTT might provide new leads for therapeutic strategies to exclusively target mHTT. A selective DUB inhibitor that improves mHTT poly-ubiquitination at its N-terminus might increase the steady-state ubiquitination of mHTT, thereby increasing the degradation of mHTT by the UPS. Indeed, several DUBs have been linked with HD, and two of them, OTULIN and USP19, seem to interfere with proteasomal degradation of mHTT (Figure 2), and might be attractive targets for DUB inhibitor development. Alternatively, compound screens with libraries of DUB inhibitors could lead to selective compounds that modulate mHTT ubiquitination and turnover, or indirectly by modifying involved ubiquitinating enzymes themselves. Inhibiting the DUB ATXN3 would counteract E3 ligase PRKN autoubiquitination (Durcan et al., 2012). Since ATXN3 and the E3 ligase CHIP are both activated by mono-ubiquitination (Todi et al., 2009; Scaglione et al., 2011) preventing their deubiquitination may preserve their activity in HD, which might be especially interesting for CHIP as it can facilitate mHTT degradation *via* the proteasome (Figure 2). While no DUB inhibitors have been described concerning HD, some DUB inhibitors showed disease-modifying effects in other neurodegenerative diseases. The USP7 inhibitor HBX41108 reduced toxicity in an ALS cell model (Zhang et al., 2020). IU1, a small molecule inhibitor of USP14, increased proteasome activity and proteasomal degradation of TAU, a hallmark protein in AD (Boselli et al., 2017). Despite these promising effects on AD pathology, USP14 inhibition may accelerate HD pathology since this DUB is associated with improved HTT clearance (Figure 2). The discrepancy between these neurodegenerative disorders underscores the importance of research on the effects of DUB inhibitors in HD.

Next to manipulating activity of ubiquitin ligases and DUBs towards mHTT, the degradation of mHTT could be improved using protein-targeting chimeras (PROTACs). PROTACs consist of a target protein-binding site and a binding motif for E3s, connected by a linker. By binding to an E3 and a protein of interest, PROTACs improve the ubiquitination of their target, which results in improved degradation by the UPS (Sakamoto et al., 2001; Békés et al., 2022). Several PROTACs were developed to treat AD and PD (Yao et al., 2022). In addition, Tomoshige and colleagues have shown the effectiveness of PROTACs on improved mHTT clearance in HD cells (Tomoshige et al., 2017; Tomoshige et al., 2018). Unfortunately, PROTACs still face many limitations, such as blood-brain barrier permeability, brain region-specific localization and the need for frequent administration (Farrell and Jarome, 2021). To make their PROTAC better suitable for delivery to the central nervous system, the HTT-targeting PROTAC was converted into a brain-permeable hydrophobic tag, which efficiently lowered HTT levels (Hirai et al., 2022). Another concern of PROTACs is that they need a functional UPS system, which is often impaired in neurodegenerative disorders (Thibaudeau et al., 2018). A possible solution might be to combine PROTAC therapy with proteasome activators (Leestemaker et al., 2017), which would selectively target mHTT, improve its ubiquitination, and activate the proteasome.

## Supplementary material

SUPPLEMENTARY TABLE S1 Detailed overview of effects of ubiquitin-modifying enzymes on cellular mHTT and HD pathology.
