# Supplementary material for: Ubiquitin-modifying enzymes in Huntington’s disease
Source: Front Mol Biosci. 2023 Feb 8;10:1107323. doi: 10.3389/fmolb.2023.1107323 (PMC10013475; doi:10.3389/fmolb.2023.1107323)
Supplement: Supplementary file 1 [file Table1.DOCX]

|  |  | **Subcellular localization** | **Model** | **Q-length** | **Enzyme levels in HD** | **Interaction (int) / co-localization (loc)** | **Enzyme modulation** | **Pathology** | **IBs** | **HTT protein levels mono/polymer** | **Ubiquitination/**  **degradation** | **ref** |
| --- | --- | --- | --- | --- | --- | --- | --- | --- | --- | --- | --- | --- |
| E1 | **UBA1** | Cytosol, nucleoplasm | Mouse | HD CAG140 KI | ↓ (cortex and stiatum) |  | Inh. PYR41 |  |  | ↑ (Poly) |  | Wade et al., 2014 |
|  |  |  |  | flHTT(Q120) |  |  | Inh. PYR41 |  |  | ↑ (Poly) |  |  |
| E2 | **HIP2** | Cytosol, plasma membrane | *in Vitro* | nHTT(Q44) | ↑ (cortex and stiatum) | Int (*in Vitro* binding assay) |  |  |  |  |  | Kalchman et al., 1996 |
|  |  |  | SH-SY5Y | nHTT(Q43)-GFP |  | Loc (dying cells; confirmed in postmortem brain tissue) | OE |  | No effect |  |  | de Pril et al., 2007 |
|  |  |  |  |  |  |  | OE mutant | Less † | ↓ |  |  |  |
|  |  |  |  |  |  |  | RNAi | Less † | ↓ |  |  |  |
|  |  |  | HD patient iPSCs | HTT(Q97/Q180) |  |  | RNAi |  | No effect |  |  | Koyuncu et al., 2018 |
|  | **UBE2W** | Nucleoli | HEK293 | nHTT(Q103)-GFP |  |  | OE |  | ↑(number/size) | No change (Mono/Poly) |  | B. Wang et al., 2018 |
|  |  |  |  |  |  |  | OE mutant^1^ |  | ↓(number/size) | No change (Mono); ↓(Poly) |  |  |
|  |  |  |  |  |  |  | OE mutant^2^ |  | ↓(number/size) | ↑ (Mono); ↓ (Poly) |  |  |
|  |  |  | Mouse primary neurons | nHTT(Q92)-EGFP |  |  | KO | Less † | ↓ | ↓ (Total levels; also wtHTT) |  |  |
|  |  |  | Mouse | HdhQ200 |  |  | KO | No effect (striatal dysfunction) | No effect (number/size) | ↑ (Mono); ↓ (Poly) | No change in Ubiquitination |  |
| E3 | **UBE3A** | Cytosol, nucleoplasm | Mouse | flHTT(Q140) | ↓ (cortex and stiatum, during aging) | Int/Loc | OE |  | ↓ |  | K63 ubiquitination ↓ | Bhat et al., 2014 |
|  |  |  | HEK293 | nHTT(Q120/Q150) |  | Int/Loc | OE |  |  | ↓ (Mono) | Proteasomal degradation ↑ |  |
|  |  |  |  | flHTT(Q120) |  |  | RNAi |  |  | ↑ (Poly) |  |  |
|  |  |  | Mouse | R6/2 (~Q150) |  | Loc (nucleus) |  |  |  |  |  | Maheshwari et al., 2012 |
|  |  |  | N2a | nHTT(Q150)-EGFP |  | Loc |  |  |  |  |  | Mishra et al., 2009 |
|  |  |  | N2a | nHTT(Q150)-EGFP | ↑ (mRNA) | Int/Loc | OE | Less † | ↓ | ↓ (Poly) |  | Mishra et al., 2008 |
|  |  |  |  |  |  |  | OE mutant |  | No effect |  |  |  |
|  |  |  |  |  |  |  | RNAi |  | ↑ | ↑ (Poly) |  |  |
|  |  |  | Mouse | R6/1 (~Q120) |  | Loc |  |  |  |  |  |  |
|  |  |  | Mouse | R6/2 (~Q150) |  |  | KO | More pathology | ↑ |  | Aggregate ubiquitination ↓ | Maheshwari et al., 2014 |
|  |  |  | HD patient iPSCs | HTT(Q97/Q180) |  |  | RNAi |  | No effect |  |  | Koyuncu et al., 2018 |
|  | **UBR5** | Cytosol, nucleoplasm | HD patient iPSCs | flHTT(Q97/Q180) |  |  | RNAi |  | ↑ | ↑ (Mono) | proteasome mediated | Koyuncu et al., 2018 |
|  |  |  | HEK293 | nHTT(Q100) |  |  | OE |  | ↓ | ↓ (Mono) | Ubiquitination ↑; proteasome mediated |  |
|  |  |  |  |  |  |  | OE mutant |  | No effect | No effect |  |  |
|  | **WWP1** | Cytosol, Golgi, plasma membrane | Mouse | R6/2 (~Q150) | ↑ | Loc (filtertrap assay) |  |  |  |  |  | Lin et al., 2016 |
|  |  |  | N2a | cherry-nHTT(Q160) | ↑ | Loc | OE | More † | ↑ | ↑ (Mono) | K63 ubiquitination ↑ |  |
|  |  |  |  |  |  |  | RNAi | Less † | ↓ | ↓ (Mono) | Ubiquitination ↓; proteasome mediated |  |
|  | **HACE1** | ER, nuclear bodies | STHdh | flHTT(Q111) | ↓ (mRNA) |  | OE | Less sensitive to oxidative stress |  |  | Sensitivity to oxidative stress independent of E3 ligase activity | Rotblat et al., 2014 |
|  |  |  | Human brain | Q20±3 (short); Q27±4 (long) | ↓ (stratium, no change cortex) |  |  |  |  |  |  |  |
|  |  |  | Mouse | YAC128 |  |  | KO | More pathology |  |  |  | Ehrnhoefer et al., 2018 |
|  | **TRAF6** | Mitochondria, nucleoli | HEK293 | nHTT(Q60/Q150)-GFP |  | Int/Loc (also wtHTT) | OE |  | ↑(number/size) | No effect | Ubiquitination ↑ (Lys6,Lys27,Lys29; also wtHTT) | Zucchelli et al., 2011 |
|  |  |  |  |  |  |  | OE mutant |  | No effect |  | No change in Ubiquitination |  |
|  |  |  | Mouse | *Hdh*Q111 |  | Int |  |  |  |  |  |  |
|  |  |  | Human brain | Q22±9 (short); Q42±1 (long) | ↑ (protein and mRNA) | Int/loc |  |  |  |  |  |  |
|  | **PJA1** | Nucleoplasm, nucleoli | N2a | nHTT(Q83)-DsRed | ↓ (protein and mRNA; also wtHTT) |  | RNAi |  | ↑ | ↑ (Mono) |  | Ghosh et al., 2021 |
|  |  |  | HEK293 | nHTT(Q83)-DsRed |  | Int/Loc (also wtHTT) | OE |  | ↓ | ↓ (Total levels; wtHTT) |  |  |
|  |  |  | Differentiated 1464R neurons | nHTT(Q74)-DsRed |  | Int | OE |  | ↓ | ↓ (Poly) |  | Watabe et al., 2022 |
|  |  |  |  |  |  |  | OE mutant |  |  | No effect |  |  |
|  |  |  | Human brain |  |  | None |  |  |  |  |  |  |
|  | **UHRF2** | Nucleoplasm | HeLa | NLS-nHTT(Q72)-GFP |  |  | OE |  |  | No effect (Mono); ↓ (Poly) | Ubiquitination ↑ (Lys6,Lys27,Lys29; also wtHTT) | Iwata et al., 2009 |
|  |  |  |  |  |  |  | OE mutant |  |  | No effect |  |  |
|  |  |  |  | NLS-nHTT(Q46)-GFP |  |  | OE |  |  |  | Half-life ↓ |  |
|  |  |  |  |  |  |  | OE mutant |  |  | No effect |  |  |
|  |  |  |  | NLS-nHTT(Q97)-GFP |  | Loc (at rim) | OE | Less † |  |  |  |  |
|  |  |  |  | NLS-nHTT(Q72)-GFP |  |  | RNAi | More † | ↑ | ↑ (Poly) |  |  |
|  |  |  | Mouse | R6/2 (~Q150) |  | Loc |  |  |  |  |  |  |
|  |  |  | Mouse primary neurons | NLS-nHTT(Q97)-GFP |  |  | OE | Less neurotoxicity |  |  |  |  |
|  |  |  |  |  |  |  | RNAi | More neurotoxicity |  |  |  |  |
|  | **San1p** - closest yeast homolog or UHRF-2 | Nucleus, cytoplasm¶ | S. cerevisiae | NLS-nHTT(Q97)-GFP |  |  | OE |  |  | ↓ (Total levels; also wtHTT) |  |  |
|  |  |  |  |  |  |  | OE mutant |  |  | No effect |  |  |
|  |  |  | HeLa | NLS-nHTT(Q72)-GFP |  |  | OE |  | ↓ | ↓ (Poly) |  |  |
|  |  |  |  |  |  |  | OE mutant |  | No effect | No effect |  |  |
|  |  |  |  | NLS-nHTT(Q46)-GFP |  |  | OE |  |  |  | Half-life ↓ |  |
|  |  |  |  |  |  |  | OE mutant |  |  | No effect |  |  |
|  |  |  |  | NLS-nHTT(Q97)-GFP |  |  | OE | Less † |  |  |  |  |
|  | **HRD1** | Nucleoplasm, ER, plasma membrane | HEK293 | FLAG-nHTT(Q138) | ↑ (protein; no effect mRNA) | Int (also wtHTT) | OE |  | ↓ |  | Half-life ↓ | Yang et al., 2007 |
|  |  |  |  |  |  |  | OE mutant |  |  |  | No change in half-life |  |
|  |  |  |  |  |  |  | RNAi |  |  | ↑ (Mono) |  |  |
|  |  |  | SH-SY5Y | GFP-nHTT(Q47/Q103) |  |  | OE |  |  | ↓ (Mono) | Ubiquitination ↑; Half-life ↓ |  |
|  |  |  |  |  |  |  | OE mutant |  |  | No effect | No change in half-life |  |
|  |  |  |  | GFP-nHTT(Q72) |  | Loc | OE | Less † | ↓ | ↓ (Mono/Poly) |  |  |
|  |  |  |  |  |  |  | OE mutant | No effect |  |  |  |  |
|  | **PRKN** | Nuclear speckles, cytosol | Patient skin fibroblasts | HTT(Q68/Q86) | ↑ (protein and mRNA) |  |  |  |  |  |  | Aladdin et al., 2019 |
|  |  |  | Mouse | YAC72 |  | Int/Loc |  |  |  |  |  | Tsai et al., 2003 |
|  |  |  | Human brain |  |  | Loc |  |  |  |  |  |  |
|  |  |  | Mouse | R6/1 (~Q120) |  |  | KD | More pathology; More † (striatum) | ↓(striatum); No effect in hippocampus |  | Autophagy (LC3II/LC3I) ↑ | Rubio et al., 2009 |
|  | **HOIP** | Cytosol | SH-SY5Y | nHTT(Q97)-GFP |  | Loc | OE |  | ↓ |  | Ubiquitination (M1) ↑; proteasome mediated | Well et al., 2019 |
|  |  |  |  |  |  |  | OE mutant |  | ↑ |  |  |  |
|  |  |  |  | nHTT(Q97) |  |  | RNAi | More † | ↑ |  |  |  |
|  |  |  | HEK293 | nHTT(Q60) |  | Int | OE |  |  |  | M1 ubiquitinationof soluble HTT ↑ |  |
|  |  |  |  | nHTT(Q60) 3R |  |  | OE |  |  |  | M1 ubiquitination of soluble HTT↓ |  |
|  |  |  |  | nHTT(Q97) |  |  |  |  |  |  | M1 ubiquitination of insoluble HTT |  |
|  |  |  | Mouse | R6/2(Q150) | ↓ (mRNA; striatum) |  |  |  |  |  | M1 ubiquitin colocalizes with Ibs |  |
|  |  |  | Human brain |  | ↓ | Loc |  |  |  |  | M1 ubiquitin colocalizes with Ibs |  |
|  | **CHIP** | Cytosol, nucleoplasm | Cos-7 | GFP-nHTT(Q82) |  |  | OE |  | ↓(number/size) | ↑ (Mono) | No change in degradation | Miller et al., 2005 |
|  |  |  |  |  |  |  | OE mutant^2^ |  |  | No effect |  |  |
|  |  |  | N2a | nHTT(Q150)-EGFP |  | Int/Loc | OE | Less † | ↓ |  | Ubiquitination ↑ | Jana et al., 2005 |
|  |  |  |  |  |  |  | OE mutant | No effect | No effect |  | No change in ubiquitination |  |
|  |  |  | Neurons | nHTT(Q130) | No change | None |  |  |  |  |  | Zhao et al., 2017 |
|  |  |  | Astrocytes | nHTT(Q130) | ↑ | Int/loc | RNAi |  |  | ↑ (Mono); ↑ (Poly) |  |  |
|  |  |  | Mouse (region high in neurons) | HD Q140 |  | None |  |  |  |  |  |  |
|  |  |  | Mouse (region high in astrocytes) | HD Q140 |  | Loc | RNAi |  |  |  | Decreased K48-Ub |  |
|  |  |  | Drosophila | nHTT(Q128) |  |  | OE | Less pathology |  |  |  | Al-Ramahi et al., 2006 |
|  | **SKP1** | Cytosol, nucleoplasm | Mouse | R6/2 (~Q150) | ↓ (cortex and cerebellum) |  |  |  |  |  |  | Bhutani et al., 2012 |
|  |  |  | Drosophila | nHTT(Q93) |  |  | RNAi | More pathology |  |  |  |  |
|  | **CUL1** | Nucleoplasm, nucleoli | Mouse | R6/2 (~Q150) | ↓ (cortex and cerebellum) | None |  |  |  |  |  |  |
|  |  |  | N2a | nHTT(Q150)-GFP | ↓ | None | Neg. OE* |  | ↑ |  |  | Bhutani et al., 2012 |
|  |  |  | Drosophila | nHTT(Q93) | ↓ (protein and mRNA) |  | RNAi | More pathology |  |  |  |  |
| DUB | **ATXN3** | Nucleoplasm, nucleoli, plasma membrane | Mouse | HdhQ200 |  | None | KO | More pathology | No effect | No effect | No change in ubiquitination | Zeng et al., 2013 |
|  |  |  | Mouse | zQ175 |  | Loc |  |  |  |  |  | Gao et al., 2019 |
|  |  |  | SH-Sy5Y | endogenous HTT |  | Int |  |  |  |  |  |  |
|  |  |  | PC12 | Myc-wtHTT-Q148 |  | Int |  |  |  |  |  |  |
|  | **OTULIN** | Mitochondria, plasma membrane | SH‐SY5Y | nHTT(Q97) |  |  | RNAi | Less † |  |  | Linear ubiquitination | van Well et al., 2019 |
|  | **YOD1** | Nucleoplasm, plasma membrane, cytosol | SH‐SY5Y | nHTT(Q74)-GFP | ↑ |  | OE | Less † |  | ↓ (Mono) | Improved degradation | Tanji et al., 2018 |
|  |  |  |  |  |  |  | OE mutant | No effect |  |  |  |  |
|  |  |  | Human brain |  |  | None |  |  |  |  |  |  |
|  | **Usp7** | Nucleoplasm, nuclear bodies | Mouse | zQ175 |  | Int |  |  |  |  |  | Pluciennik et al., 2021 |
|  |  |  | HD patient iPSCs | HTT(Q109) |  | Int |  |  |  |  |  |  |
|  | **Usp12** | Nucleoplasm | Primary neurons | GFP-nHTT(Q138) |  | Loc | OE | Less † |  |  | No change in half-life; autophagy mediated | Aron et al., 2018 |
|  |  |  |  |  |  |  | RNAi | More † |  |  |  |  |
|  |  |  |  |  |  |  | OE mutant | Less † |  |  | No change in half-life |  |
|  |  |  | HD patient iPSCs | HTT(Q109) |  |  | OE | Less † |  |  |  |  |
|  |  |  | Drosophila | nHTT(Q93) |  |  | OE | Less pathology |  |  |  |  |
|  |  |  |  |  |  |  | RNAi | More pathology |  |  |  |  |
|  |  |  | HD patient line | HTT(Q53) |  |  | OE | Less † |  |  |  |  |
|  |  |  |  |  |  |  | OE mutant | Less † |  |  |  |  |
|  | **USP14** | Plasma membrane, cytosol | PC6.3 | nHTT(Q120) | Changed localization |  | OE | Less † | ↓ | ↓ (Poly) | Proteasome mediated + increased ER stress and cell death | Hyrskyluoto et al., 2014 |
|  |  |  |  |  |  |  | OE mutant |  | No effect |  |  |  |
|  |  |  | HeLa | nHTT(Q103) |  | Int (also wtHTT) | OE |  | ↓ | ↓ (Poly) |  |  |
|  |  |  |  |  |  |  | RNAi |  | ↑ (n.s.) |  |  |  |
|  |  |  | Mouse | BACHD | No change |  |  |  |  |  |  |  |
|  | **USP19** | ER membrane, cytosol¶ | HEK293 | nHTT(Q100)-GFP |  |  | OE | More pathology | ↑ | ↓ (Mono) | Increased deubiquitination | He et al., 2016 |
|  |  |  |  |  |  |  | OE mutant | No effect | No effect | no effect |  |  |
|  |  |  |  |  |  |  | RNAi |  |  | ↓ (Mono) |  |  |
|  |  |  | HEK293 | nHTT(Q100)-GFP |  |  | OE |  | ↑ |  | Via HSP90 | He et al., 2017 |

*Table notes. All enzymes are expressed in brain tissue. Localization data is obtained from The Human Protein Atlas. If no localization data was available in The Human Protein Atlas, information was obtained from COMPARTMENTS and marked with ¶. OE = Overexpression. OE mutant = Overexpression ligase/catalytic dead mutant. OE mutant^1^ = Substrate binding mutant. OE mutant^2^ = Chaperone interaction mutant. Neg. OE^§^ = Dominant negative overexpression.* *† = Death.*
